# Dexamethasone-induced immunosuppression: mechanisms and implications for immunotherapy

**DOI:** 10.1186/s40425-018-0371-5

**Published:** 2018-06-11

**Authors:** Amber J. Giles, Marsha-Kay N. D. Hutchinson, Heather M. Sonnemann, Jinkyu Jung, Peter E. Fecci, Nivedita M. Ratnam, Wei Zhang, Hua Song, Rolanda Bailey, Dionne Davis, Caitlin M. Reid, Deric M. Park, Mark R. Gilbert

**Affiliations:** 1 Neuro-Oncology Branch, CCR, NCI, National Institutes of Health, 37 Convent Dr. Bldg. 37, Rm. 1142B, Bethesda, MD 20892 USA; 2 Department of Neurosurgery, Duke University Medical Center, Durham, NC USA

**Keywords:** Corticosteroids, Immunotherapy, Glioma, Checkpoint blockade, Dexamethasone

## Abstract

**Background:**

Corticosteroids are routinely utilized to alleviate edema in patients with intracranial lesions and are first-line agents to combat immune-related adverse events (irAEs) that arise with immune checkpoint blockade treatment. However, it is not known if or when corticosteroids can be administered without abrogating the efforts of immunotherapy. The purpose of this study was to evaluate the impact of dexamethasone on lymphocyte activation and proliferation during checkpoint blockade to provide guidance for corticosteroid use while immunotherapy is being implemented as a cancer treatment.

**Methods:**

Lymphocyte proliferation, differentiation, and cytokine production were evaluated during dexamethasone exposure. Human T cells were stimulated through CD3 ligation and co-stimulated either directly by CD28 ligation or by providing CD80, a shared ligand for CD28 and CTLA-4. CTLA-4 signaling was inhibited by antibody blockade using ipilimumab which has been approved for the treatment of several solid tumors. The in vivo effects of dexamethasone during checkpoint blockade were evaluated using the GL261 syngeneic mouse intracranial model, and immune populations were profiled by flow cytometry.

**Results:**

Dexamethasone upregulated CTLA-4 mRNA and protein in CD4 and CD8 T cells and blocked CD28-mediated cell cycle entry and differentiation. Naïve T cells were most sensitive, leading to a decrease of the development of more differentiated subsets. Resistance to dexamethasone was conferred by blocking CTLA-4 or providing strong CD28 co-stimulation prior to dexamethasone exposure. CTLA-4 blockade increased IFNγ expression, but not IL-2, in stimulated human peripheral blood T cells exposed to dexamethasone. Finally, we found that CTLA-4 blockade partially rescued T cell numbers in mice bearing intracranial gliomas. CTLA-4 blockade was associated with increased IFNγ-producing tumor-infiltrating T cells and extended survival of dexamethasone-treated mice.

**Conclusions:**

Dexamethasone-mediated T cell suppression diminishes naïve T cell proliferation and differentiation by attenuating the CD28 co-stimulatory pathway. However, CTLA-4, but not PD-1 blockade can partially prevent some of the inhibitory effects of dexamethasone on the immune response.

**Electronic supplementary material:**

The online version of this article (10.1186/s40425-018-0371-5) contains supplementary material, which is available to authorized users.

## Background

Immunotherapy is emerging as a promising anti-cancer treatment and is now part of the standard of care for certain advanced cancers including melanoma and non-small cell lung carcinoma [1]. Encouraging results from recent studies suggest that intracranial lesions located beyond the blood-brain barrier may also be targeted by the immune system [2–5]. However, patients with intracranial lesions are frequently provided corticosteroids before commencing immunotherapy to combat cerebral edema and reduce symptom burden. Corticosteroids are also first-line agents against immune-related adverse events (irAEs) that may develop during or following immunotherapy, particularly checkpoint blockade [6, 7]. To date, it remains unclear how steroids impact adaptive anti-tumor immunity [8, 9], and whether the effects of corticosteroids on immune response differs if they are administered prior to initiation of immune therapy or after an immune response has been generated.

Although a subset of patients receiving corticosteroids while undergoing treatment with immunce checkpoint inhibitors have achieved clinical benefit, there are concerns that they exhibit poorer response to checkpoint blockade [10–12]. Other studies found that corticosteroids do not negatively impact overall survival of patients on immunotherapy involving CTLA-4 blockade [13–18]. However, these studies were not powered to specifically address the impact of corticosteroids on immunotherapeutic response. Given the varied reports, the immunosuppressive effects of corticosteroids require further interrogation, particularly understanding the impact of this treatment when administered at the initiation of checkpoint blockade therapy, a situation likely to be common in patients with intracranial malignancies.

Exogenous corticosteroids are toxic to immature T cells, including thymocytes and acute lymphoblastic leukemia blasts [19, 20]. However, less is known about the impact of corticosteroids on mature lymphocytes. Whereas corticosteroids have been shown to suppress IL-2-mediated T cell proliferation and cytokine production; they can also induce expression of the pro-survival receptor IL-7Rα [21, 22]. T cell reactivity against cytomegalovirus, a potential antigen in glioblastoma [23, 24], was not impaired in patients with glioblastoma actively receiving or previously on dexamethasone [25]. Further, pre-operative corticosteroids did not reduce the density of tumor-infiltrating lymphocytes in patients with brain metastases [26]. These observations suggest that antigen-stimulated memory T cells, a critical population for patients receiving immunotherapy, may be resistant to corticosteroid exposure.

Here, we interrogated the impact of dexamethasone on T cell proliferation, differentiation, and cytokine production using human T cells and a murine glioblastoma model. We demonstrated that dexamethasone attenuates the CD28 co-stimulatory pathway by upregulating CTLA-4, thereby severely inhibiting naïve T cell proliferation and differentiation. This inhibition can be overcome in vivo by using a CTLA-4 neutralizing antibody, which extended survival in a murine syngeneic glioblastoma model. These findings have important implications for corticosteroid use with immune checkpoint blockade, particularly in patients with central nervous system tumors where corticosteroids are regularly utilized to mitigate peritumoral edema.

## Methods

### Preparation of Dynabeads®M-450 Tosylactivated beads

Dynabeads**®** M-450 Tosylactivated beads (Invitrogen by Life Technologies) were coupled with 50 μg of protein according the manufacturer’s guidelines and as previously shown [27]. Isotype beads were coupled with 10% Purified NA/LE Mouse IgG2a, k isotype control (BD Bioscience), 30% Ultra-LEAF Purified Mouse IgG1, k isotype control (Biolegend) and 60% Ultra-LEAF Purified Human IgG1 isotype control antibody (Biolegend). Stimulatory beads were designed using 5% Purified NA/LE Mouse anti-human CD3 (HIT3a; BD Biosciences), 30% Ultra-LEAF Purified Human IgG1 Isotype control antibody (Biolegend), 15% Ultra-LEAF Purified anti-human CD28 antibody (CD28.2; Biolegend) or Recombinant human B7–1/CD80-Fc Chimera Protein, CF (R&D Systems). Beads were stored in Ca^2+^ and Mg^2+^ free PBS with 0.1% BSA and 2 mM EDTA pH 7.4 at 4 **°**C.

### T-cell preparation, culture and treatment

Healthy donor leukapheresis packs were obtained from the NIH blood bank (protocol 99-CC-0168). T cells were negatively selected using an EasySep Human T cell isolation kit (Stemcell Technologies) and cryopreserved in 90% FBS and 10% DMSO until use. Cells were thawed in a 37°C water bath and cultured overnight in RPMI1640 medium containing 10% fetal bovine serum, 1% penicillin-streptomycin-glutamine, 1% MEM non-essential amino acids solution, 15 mM HEPES, 1 mM sodium pyruvate and 55 μM 2-mercaptoethanol. Cells were plated at 1*10^5^/200 μl in 96-well round-bottom plates with M-450 Tosylactivated beads. Dexamethasone was purchased from Sigma Aldrich (D4902) and dissolved in DMSO. Niviolumab and ipilimumab F(ab’)_2_ were used to block PD-1 and CTLA-4, respectively. Ipilimumab F(ab’)_2_ was created using a Pierce F(ab’)_2_ Preparation Kit per the manufacturer’s instructions (ThermoFisher Scientific, MA, USA). Cells were incubated at 37 **°**C in 20% O_2,_ and 5% CO_2_ for four days for proliferation analyses and two days for Western blot and qPCR analyses.

### Flow cytometry analysis

αCD152-PE (BNI), αCD8α-Pacific-Blue or Alexa 488 (RPA-T8), αCD4-APC (RPA-T4), αCD197-Brilliant Violet 605 (G043H7), and αCD45RO-Brilliant Violet 785(UCHL1) were purchased from BioLegend. Anti-CD4-Brilliant UV 496 (SK3) and anti-CD279-APC (clone EH12.2.2H7) were purchased from eBioscience. Cells were stained for 30 min at room temperature, rinsed with FACS buffer (1% BSA and 0.01% sodium azide in PBS), fixed with 4% paraformaldehyde (Alfa Aesar, WA, USA), and resuspended in FACS buffer for flow cytometry analysis. Cell Cycle analysis was conducted using a Click-iT® Edu Flow Cytometry Assay Kit (Invitrogen, Carlsbad, CA, USA) per the manufacturer’s instructions. Cell apoptosis was investigated using Annexin V-Pacific Blue (Biolegend, San Diego, CA, USA) and propidium iodide (Sigma Aldrich, MO, USA). Data were acquired on a BD LSR Fortessa SORP II or LSR Fortessa X50, analyzed with Flowjo version 9.9.4 and SPICE version 5.35.

### Western blot analysis

Isolated human T cells were collected after 48 h of stimulation and lysed in RIPA buffer with EDTA- free protease inhibitor cocktail set III (EMD Millipore, Billerica, Massachusetts, USA). Pierce BCA protein assay kit (Thermo Fisher Scientific, Rockford, IL, USA) was used to determine protein concentration. Samples were separated by SDS-PAGE (Bio-Rad) and transferred onto 0.2 μm pore size polyvinylidene fluoride membranes (PVDF) (Invitrogen, Carlsbad, CA, USA). The following antibodies were purchased from Cell Signaling: cleaved caspase 3 (5A1E), p27kip (2552 s), cyclin D3 (DCS22), CDK4 (D9G3E). Anti-CTLA-4 (EPR1476) was purchased from Abcam. The bands were detected by Super Signal West Pico chemiluminescence reagent (Pierce, Rockford, IL, USA). Antibodies against β-actin (AG74) or GAPDH (FL-335) were used as internal standards.

### RT-PCR

Total RNA was extracted using Trizol reagent (Invitrogen). Reverse transcription was performed using the Superscript III First-Strand Synthesis System (Invitrogen) with oligo dT. Subsequent RT-PCR was performed using SYBR green reagent from ABI and run on an ABI Quant Studio7. β-actin was used as a control to normalize gene expression. Primers and conditions used for RT-PCR are listed in the table below.

**Table:** 

Gene	Primer Sequence	T_m_(°C)
IFNγ	F- 5’ TCGGTAACTGACTTGAATGTCCA 3′ R- 5’ TCGCTTCCCTGTTTTAGCTGC 3’	60
IL-2	F- 5’ AACTCCTGTCTTGCATTGCAC 3′ R- 5’GCTCCAGTTGTAGCTGTGTTT 3’	60
CTLA-4	F- 5’ CATGATGGGGAATGAGTTGACC 3′ R- 5’ TCAGTCCTTGGATAGTGAGGTTC 3’	60
β-actin	F-5’ CCACACTGTGCCCATCTAC 3′ R-5’ CCATCTCTTGCTCGAAGTCC 3’	60

### Murine studies

All experiments were approved by the NCI-Bethesda Animal Care and Use Committee. Six to 8 week-old female albino C57BL/6 mice were purchased from Jackson laboratories (Bar Harbor, ME). For intracranial tumor implantation, mice were injected with 1*10^3^ GL261 cells that were stably transduced with a firefly luciferase-mCherry lentiviral vector. Water-soluble dexamethasone (D2915, Sigma Aldrich) was administered at 1 mg/kg/day by oral gavage. The non-toxic solubilizer, 2-hydroxypropyl-beta-cyclodextrin (H-107, Sigma Aldrich) was dissolved with water and matched in concentration to water-soluble dexamethasone for vehicle control. InvivoMab anti-mouse CTLA-4 (BE0131, BioXCell) or InVivoMab polyclonal Syrian hamster IgG isotype antibody (BE0087, BioXCell) were administered by intraperitoneal injection. Tumor was detected by luminescence imaging and analyzed with LivingImage Software.

### Statistical analysis

Statistical Analysis was performed using GraphPad Prism 7.0 software. Data are expressed as the mean +/− standard deviation and statistical significance evaluated by two-tailed Student’s t-test. *P*-values < 0.05 were considered significant.

## Results

### Dexamethasone impairs proliferation of mature T cells

T cells require two signals for an effective proliferative response; the first signal arises when a T cell receptor (TCR) binds its cognate peptide:MHC complex, resulting in an intracellular signaling cascade through CD3. The second, co-stimulatory signal, is received when CD28 on T cells binds either CD80 or CD86 on antigen-presenting cells (APCs) [28]. Here, T cells isolated from healthy donor peripheral blood samples were stimulated with an αCD3 antibody for signal 1 and either an αCD28 antibody or recombinant CD80 protein for co-stimulation [27]. Recombinant CD80 permitted us to dissect the role of extracellular molecular interactions between CD80 and its multiple binding partners on T cells. These partners include the positive co-stimulatory molecule, CD28, and negative co-stimulatory molecules, such as CTLA-4 and PD-1.

**Fig. 1:**
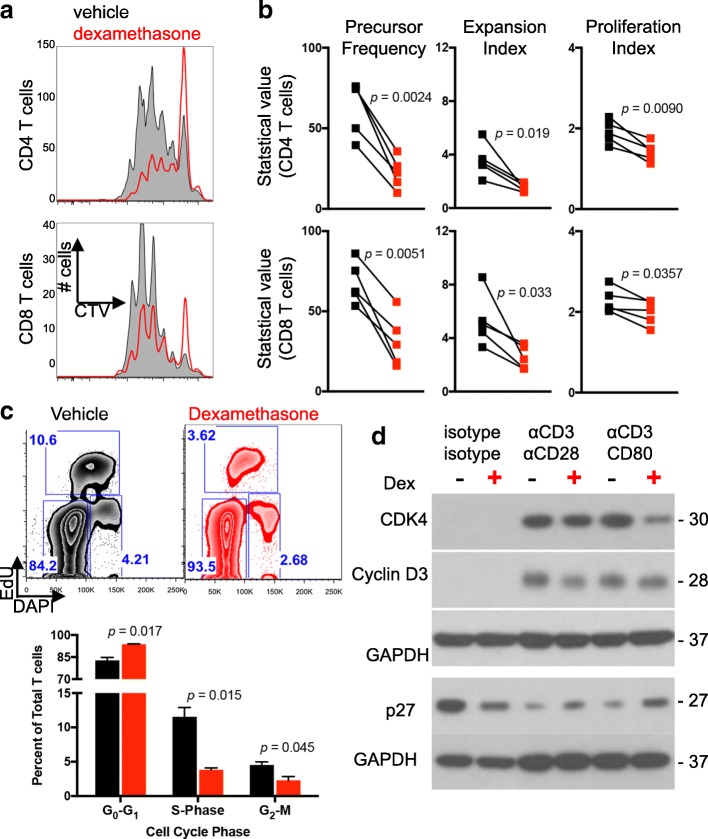
T cell proliferation is impaired by dexamethasone. Healthy donor T cells were cultured for four days with αCD3/CD80 microbeads in the presence of vehicle or dexamethasone. **a** Representative flow cytometry plots of CellTrace violet dilution. Plots were derived from gated CD4 (top row) or CD8 (bottom row) T cells. **b** Negatively-selected healthy donor T cells were stained and proliferation analyses determined by flow cytometry following four days of culture under the indicated conditions. Precursor Frequency, Expansion Index, and Proliferation Index are shown. Each symbol is the average of duplicate wells, and each paired symbol represents a different donor (*n* = 5 donors). Statistical significance was determined with a paired two-tailed T test. **c** Cell cycle analysis was performed on healthy donor T cells cultured with vehicle or dexamethasone and stimulated with αCD3/CD80 microbeads. EdU uptake and DNA content were used to identify G_0_/G_1_, S, and G_2_/M phases. Representative flow images (top) and quantification of duplicate wells are shown (bottom) from two independent experiments. **d** Lysates from healthy donor T cells incubated with the indicated microbeads and vehicle or dexamethasone were probed for the indicated proteins. GAPDH was used as a loading control and is shown for each individual blot. Data are representative of three independent experiments

Human T cells incubated with αCD3 and CD80 underwent multiple rounds of division, demonstrating that recombinant CD80 provided an efficient co-stimulatory signal under normal culture conditions (Fig. 1a). However, dexamethasone exposure caused defects in cell division for both CD4 and CD8 T cells. Cell proliferation statistics, including precursor frequency, expansion index, and proliferation index, were performed on multiple heathy donor T cells isolated from peripheral blood mononuclear cells to assess population dynamics and identify the proliferation defect [29]. Precursor frequency, defined as the cell-intrinsic probability that a cell will undergo at least one division, was diminished when T cells were exposed to dexamethasone (Fig. 1b). Similarly, dexamethasone-exposed T cells also had a reduced expansion index, a cell-extrinsic statistic used to express the fold expansion of the final cell count compared to the initial cell count (including undivided cells). Finally, the proliferation index, which represents the number of divisions that cells in the post-mitotic population have undergone, was modestly decreased by dexamethasone. Thus, cell proliferation statistics demonstrate that dexamethasone impaired the ability of CD80 co-stimulated CD4 and CD8 T cells to divide and restricted the expansion potential of these populations. In contrast, dexamethasone imposed only subtle cell division defects under all conditions in which CD28 was exclusively ligated with an αCD28 antibody (Additional file 1: Figure S1), indicating that direct ligation of CD28 can confer resistance to dexamethasone.

### Dexamethasone attenuates the CD28 signaling pathway

Increased apoptosis and cell cycle blockade can each manifest as a proliferative defect in vitro. Dexamethasone did not increase the frequency of apoptotic T cells when cultured with αCD3 and either αCD28 or CD80 co-stimulation as demonstrated by flow cytometry and Western analysis of cleaved caspase 3 (Additional file 2: Figure S2), demonstrating that cell death was not responsible for the proliferative defect. Indeed, dexamethasone has been shown to protect T cells from activation-induced cell death [30].

Cell cycle progression of CD80-costimulated T cells was next evaulated in response to dexamethasone exposure. Dexamethasone induced, on average, a 13% increase in the G_0_/G_1_ phase along with a 67% reduction of cells in S-phase and a 49% reduction in the G_2_/M phase (Fig. 1c). Dexamethasone-exposed T cells also contained increased levels of p27 protein, an inhibitor of cell cycle entry, and concurrent reductions in CDK4 and cyclin D3 protein. Together, these data demonstrate that dexamethasone blocks cell cycle entry in T cell stimulated with αCD3 and CD80.

### Co-stimulation protects T cells from the anti-proliferative effects of dexamethasone

**Fig. 2:**
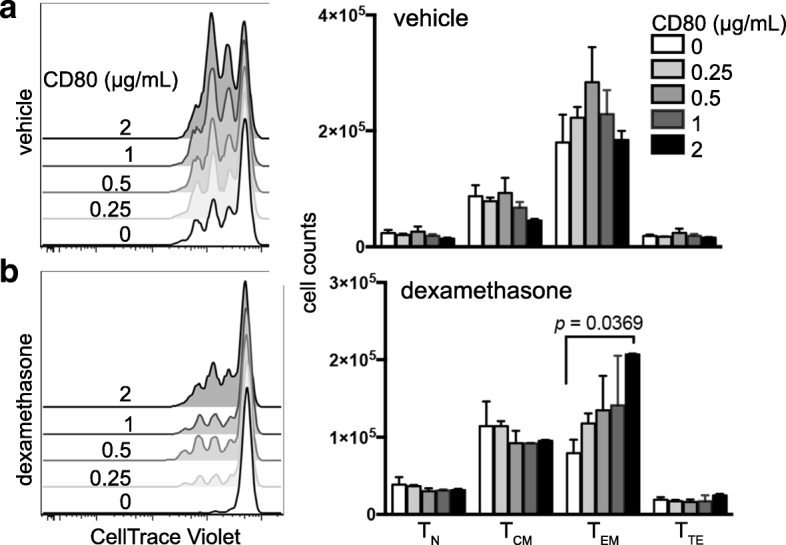
Increased co-stimulation ameliorates the inhibitory effects of dexamethasone. Negatively-selected healthy donor T cells were cultured with 5 μg/mL αCD3 and increasing concentrations of CD80 in the presence of vehicle or dexamethasone. **a-b** CD4 T cells cultured with vehicle (**a**) or dexamethasone (**b**). Flow cytometry plots showing proliferation of cells cultured with the indicated concentration of CD80 (left) and total numbers of naïve (T_N_), central memory (T_CM_), effector memory (T_EM_), and terminal effector (T_TE_) T cells following four days of culture (right) are shown. Differentiation subsets were assessed by CD45RO and CCR7 staining. Each condition was plated in duplicate, and data are representative of three independent experiments. Data were analyzed with an unpaired, two-tailed T Test

Following stimulation-induced expansion T cells may advance to a more terminally differentiated state. However, T cells cultured with dexamethasone had a greater proportion of naïve T cells (T_N_) and central memory T cells (T_CM_) and fewer effector memory T cells (T_EM_) than cells cultured with vehicle control after four days of stimulation (Additional file 3: Figure S3). Terminal effector T cells (T_TE_) were unchanged. These data indicate that dexamethasone blocked the ability of T cells to differentiate in response to αCD3/CD80 stimulation. The loss in T_EM_ numbers was reversed by increasing concentrations of CD80 (Fig. 2 and Additional file 4: Figure S4), suggesting that strong co-stimulation may protect this subset from the inhibitory effects of dexamethasone.

**Fig. 3:**
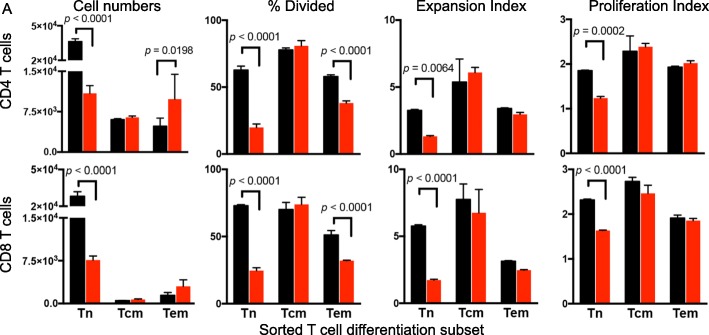
Naïve and effector memory T cells show sensitivity to dexamethasone. **a** Healthy donor T cells were sorted into T_N_, T_CM,_ and T_EM_ subsets by flow cytometry. Sorted subsets were cultured with αCD3/CD80 microbeads in the presence of dexamethasone (red) or vehicle control (black). Total cell numbers, Precursor Frequency, Expansion Index, and Proliferation Index of CD4 T cells (top) and CD8 T cells (bottom) are shown. All samples were plated in duplicate and analyzed with a paired, two-tailed T test. Data are representative of three independent experiments

To directly test the impact of dexamethasone on T cell differentiation subsets, circulating T cells from healthy donors were sorted by flow cytometry into T_N_, T_CM_, and T_EM_ subsets. The proliferative response of each was assessed in response to stimulation and exposure to vehicle or dexamethasone. Cultures from purified naïve CD4 and CD8 T cells were most severely impaired by dexamethasone, with a diminished precursor frequency, expansion index, and proliferation index (Fig. 3). In contrast, purified T_CM_ response was comparable to vehicle control. T_EM_ demonstrated reduced precursor frequency but greater cell number, suggesting that although dexamethasone impaired cell cycle entry, the cells were not lost due to apoptosis. Also, unlike T_N_, dexamethasone did not impair the expansion or proliferation index of T_EM_, demonstrating that this subset was less sensitive to dexamethasone than T_N_.

### CTLA-4 blockade protects T cells from the deleterious effects of dexamethasone

**Fig. 4:**
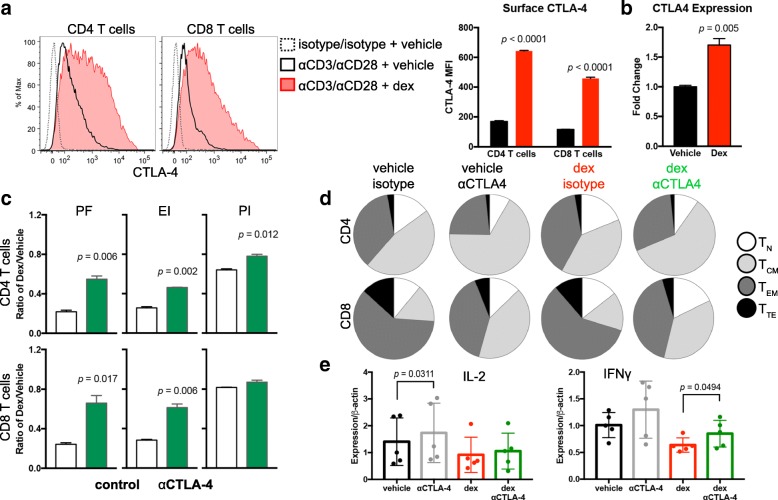
CTLA-4 blockade partially restores T cell proliferation in the presence of dexamethasone. **a** Flow cytometry analysis of CTLA-4 surface expression on CD4 (left) or CD8 (right) T cells stimulated with αCD3/αCD28 microbeads. Unstimulated (dashed line), stimulated in presence of vehicle (solid line), and stimulated in presence of dexamethasone (filled red line) are shown (left) and median fluorescence intensity (MFI) of CTLA-4-expressing T cells is quantified (right). Data are representative of four independent experiments. **b** Expression of CTLA-4 by qPCR of T cells stimulated in the presence of vehicle or dexamethasone. Data are representative of four independent experiments. **c** Healthy donor T cells stimulated for four days in the presence of vehicle or dexamethasone and with or without ipilimumab F(ab’)_2_ antibody. Proliferation analysis of CD4 T cells (top) and CD8 T cells (bottom) was performed, and the ratio of cells stimulated with dexamethasone relative to vehicle control are shown for Precursor Frequency (PF), Expansion Index (EI), and Proliferation Index (PI). All samples were plated in duplicate and the ratios were analyzed with an unpaired, two-tailed T test. Data are representative of 7 healthy donors. **d** Cells were cultured as in (**c**). The number of T cells in each differentiation group were quantified by flow cytometry and analyzed by SPICE. **e** Expression of the indicated cytokines was determined by qPCR. Five healthy donors were assayed for each condition. Each data point represents and average of triplicate wells. Data were analysed with a paired, two-tailed T test

CD80 provides a positive co-stimulatory signal to T cells when bound by CD28. However, following TCR signal transduction, CTLA-4 is translocated to the outer membrane of T cells where it can outcompete CD28 to bind CD80 and block co-stimulation [31–34]. Using flow cytometry, we confirmed that T cell stimulation led to increased extracellular CTLA-4 protein levels. In the presence of dexamethasone, however, stimulation caused a fourfold increase in surface CTLA-4 protein compared to vehicle treated (Fig. 4a) as well as an increase in CTLA-4 transcription (Fig. 4b). For these experiments, T cells were stimulated with αCD3 and αCD28 antibodies because surface CTLA-4 is internalized following ligation by CD80 [35], potentially impairing detection by flow cytometry antibodies. CTLA-4 expression was consistently found to be higher in CD4 T cells compared to CD8 T cells during dexamethasone treatment, likely rendering CD4 T cells more susceptible to cell cycle inhibition by checkpoint molecule.

We hypothesized that dexamethasone-induced CTLA-4 upregulation on T cells out-competed CD28 for the shared CD80 ligand, thereby attenuating the CD28 co-stimulatory pathway and inhibiting cell cycle entry. To test this, CTLA-4 was blocked with ipilimumab, a monoclonal anti-CTLA-4 human antibody, thereby enhancing the ability of CD28 to bind CD80. With ipilimumab treatment, dexamethasone-induced cell proliferation defects were partially restored. Precursor frequency and expansion index of dexamethasone-treated CD4 and CD8 T cells were significantly increased (Fig. 4c) as well as the proliferation index of CD4 T cells. In contrast, the proliferation index of CD8 T cells co-stimulated with CD80 was not increased with ipilimumab. T cells exposed to dexamethasone also upregulated of PD-1 during in vitro stimulation, but blocking PD-1 with nivolumab did not significantly change T cell precursor frequency (Additional file 5: Figure S5). These data demonstrate that ipilimumab specifically reversed the proliferation defects caused by dexamethasone, implicating CTLA-4 as a mechanism for the anti-proliferative activities of dexamethasone exposure in T cells.

The relative proportion of T_N_ and T_CM_ were increased in cultures exposed to dexamethasone compared to vehicle control. CTLA-4 blockade with ipilimumab led to an expansion of the T_CM_ subset, and this was recapitulated in T cells cultured with the combination of ipilimumab and dexamethasone (Fig. 4d). Blocking CTLA-4 with ipilimumab increased IFNγ, but not IL-2 transcription during dexamethasone exposure, suggesting that CTLA-4 blockade may rescue IFNγ-producing T cells inhibited by dexamethasone (Fig. 4e).

### Checkpoint blockade enhances survival of dexamethasone-treated tumor-bearing mice

**Fig. 5:**
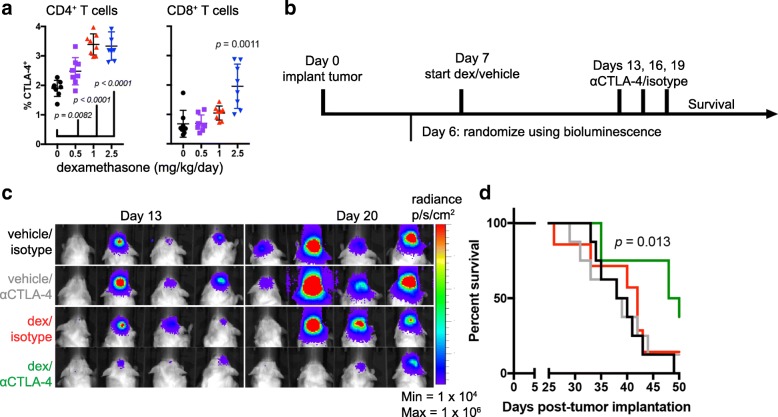
CTLA-4 blockade enhances survival of dexamethasone-treated mice. **a** CTLA-4 was measured on circulating CD4 (left) and CD8 (right) T cells 1 h following oral gavage of vehicle or the indicated concentration of dexamethasone. Each cohort contained eight mice with intracranial GL261 tumors. Vehicle and dexamethasone-treated cohorts were statistically analyzed with an unpaired two-tailed student’s T-test. **b** Schema of treatment cohorts for (**c-d**). GL261 ffluc-mCherry glioma cells were orthotopically implanted into C57BL/6 mice one week before treatment initiation. Luminescence readings were acquired 6 days following tumor implantation and weekly thereafter. Mice were treated with vehicle or dexamethasone as indicated. CTLA-4 blocking antibody or isotype control were administered on days 13, 16, and 19 following tumor implantation. **c** Luminescence of tumor-bearing mice at days 13 and 20 following tumor implantation. **d** Kaplan Meier survival curves of mice receiving the indicated treatments. *n* = 7 to 8 mice per cohort. Data are representative of two independent experiments

To determine if these findings translated to in vivo models, the C57Bl/6 syngeneic glioma tumor line GL261 was implanted intracranially. Consistent with the human in vitro data, dexamethasone increased the percentage of CTLA-4-expressing CD4 T cells of tumor-bearing mice in a dose-dependent manner (Fig. 5a). The percent of CTLA-4-expressing CD8 T cells also significantly increased in mice treated with the highest concentration of dexamethasone (2.5 mg/kg/day). Because dexamethasone upregulated CTLA-4, we hypothesized that CTLA-4 blockade would provide a survival advantage to dexamethasone-treated mice. GL261 tumor cells were implanted one week before commencement of dexamethasone or vehicle treatment to allow for immune surveillance and development of differentiated anti-tumor T cells. CTLA-4 blockade or isotype antibody were administered on days 13, 16, and 19 to vehicle or dexamethasone-treated mice to emulate the clinical scenerios where patients may be off or on corticosteroids before receiving immunotherapy (Fig. 5b). To ensure comparable tumor burden, mice were randomized into cohorts with equivalent tumor luminescence prior to treatment, and luminescence was measured weekly thereafter (Fig. 5c).

Single-agent treatment with CTLA-4 blocking antibody or dexamethasone did not produce a significant survival benefit compared to vehicle-treated animals in this well-established glioblastoma model. However, mice that received CTLA-4 blockade in addition to dexamethasone treatment survived significantly longer than control animals, with a median survival of 49 versus 39 days, respectively (*p* = 0.013; Fig. 5d). Here, the immune system surveyed the immunogenic GL261 luciferase-mCherry tumor for one week before dexamethasone treatment was initiated. To determine if immune priming contributed to dexamethasone resistance, this experiment was repeated to include a cohort of mice exposed to dexamethasone one day prior to tumor implantation. In this cohort, CTLA-4 blockade did not provide a survival benefit (Additional file 6: Figure S6). These data indicate that dexamethasone exposure may block a successful anti-tumor immune response if treatment occurs before anti-tumor T cells can differentiate from the T_N_ pool.

**Fig. 6:**
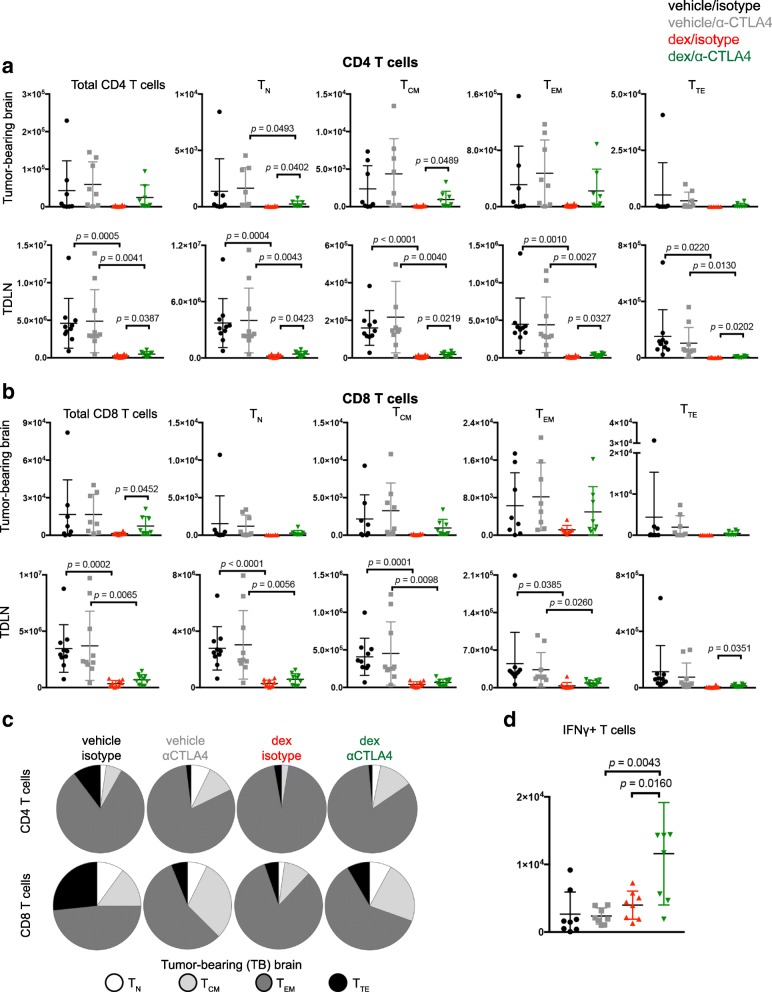
CTLA-4 blockade rescues lymphocyte defects induced by dexamethasone. GL261 ffluc-mCherry tumor-bearing mice were randomized into the indicated cohorts. Vehicle or dexamethasone treatment was initiated on day 7, and isotype or CTLA-4 blocking antibody were administered on days 13, 16, and 19 following tumor implantation. Mice were euthanized on day 23 and tissues were harvested for flow cytometry analysis. **a-b** CD4 (**a**) and CD8 (**b**) T cells were quantified along with the indicated differentiation subsets using CD44 and CD62L expression. Brains (*n* = 8) and cervical lymph nodes (*n* = 10) were collected. Data are analyzed using a unpaired students T test. **c** The relative contribution of each differentiation subset is shown for CD4 (top) and CD8 (bottom) TILs. **d** The total number of IFNγ-producing T cells were quantified from the tumor-bearing hemispheres of mice from the indicated cohorts. Data are analyzed using an unpaired students T test. *N* = 8 mice/group

The impact of dexamethasone exposure was next evaluated on lymphocyte populations from tumor-bearing mice, including tumor-infiltrating lymphocytes (TILs) and T cells in the tumor-draining cervical lymph nodes (TDLN). Mice exposed to dexamethasone possessed significantly reduced numbers of T cells in TDLNs. However, CTLA-4 blockade increased the total number of CD4 T cells in TDLN and CD8 TILs (Fig. 6a and b). The number of regulatory T cells (Treg) in the tumor-bearing brain were not significantly affected by dexamethasone or CTLA-4 blockade whereas overall numbers were reduced in TDLNs from each dexamethasone-treated cohort (Additional file 7: Figure S7A).

Dexamethasone exposure reduced the total number of T cells in the majority of T cell subsets along the differentiation spectrum in the tumor-draining lymph nodes, yet as observed in vitro, this was partially rescued by CTLA-4 blockade (Fig. 6a and b). The number of CD4 T_N_ and T_CM_ TIL subsets were also significantly elevated by CTLA-4 blockade in the dexamethasone-treated group. The relative proportion of T_CM_ TILs was increased by CTLA-4 blockade in both the vehicle and dexamethasone-treated cohorts (Fig. 6c). GBM has been reported to induce high expression of checkpoint molecules on TILs, resulting in a severe exhaustion signature [36]. Dexamethasone treatment increased the percentage of CD8 TILs that expressed Tim-3 from 2 to 12%, although it did not significantly impact other checkpoint molecules (PD-1 or Lag-3) or the frequency of TILs expressing multiple checkpoint molecules (Additional file 7: Figure S7B). Although T cells expressing checkpoint molecules were less frequent in TDLN, dexamethasone decreased the frequency of most checkpoint-expressing T cells with the exception of Tim-3^+^ CD8 T cells, which increased from 1.5 to 7.5 and 1.3 to 4.7% in isotype and CTLA-4 blockade treated mice, respectively. Finally, the number of IFNγ-producing TILs in dexamethasone-treated mice was significantly increased with CTLA-4 blockade, consistent with in vitro data (Fig. 6d). Together, these data demonstrate that CTLA-4 blockade can partially reverse the inhibitory effects of dexamethasone on T cells in vivo*,* reduce the proportion of TILs expressing checkpoint molecules, increase IFNγ-expressing TILs and extend survival of dexamethasone-treated mice bearing intracranial gliomas.

## Discussion

Corticosteroids, most commonly dexamethasone, are regularly administered to patients with intracranial tumors to combat cerebral edema and provide symptomatic relief [37]. Additionally, corticosteroids are used to treat patients who develop irAEs as a result of immunotherapy. In contrast to the frequent early use of dexamethasone for tumor-associated edema, corticosteroid use for immunotherapy-related toxicity is always given after treatment has commenced. Corticosteroids have been established as causing dose-related immunosuppression, yet the mechanisms behind this impaired immune function, particularly in the context of cancer immunotherapy, have not been defined [38]. Importantly, it is not known if corticosteroids impede the differentiation of freshly stimulated T cells or if they deplete established and already differentiated tumor-reactive lymphocytes.

In this study, the immunosuppressive effects of dexamethasone on individual T cell differentiation subsets were interrogated. T_N_ were identified as being exceptionally sensitive to dexamethasone-mediated cell cycle blockade. T_N_ are a key source of secondary anti-tumor immunity mediated by antigen spread in response to checkpoint blockade [39] and are highly sensitive to anergy imposed by expression of CTLA-4 [40]. Dexamethasone exposure strongly upregulated PD-1 and CTLA-4 transcription and protein, consistent with previous in vitro murine studies [41, 42]. The data presented here extend upon these studies to demonstrate that dexamethasone-induced CTLA-4 upregulation effectively blocked T_N_ proliferation and differentiation in both murine and human T lymphocytes. In tumor-bearing mice, this led to a loss of differentiated T cell subsets in several lymphoid tissues.

In contrast to T_N_ lymphocytes, dexamethasone exposure had much less impact on memory T cell proliferation following flow cytometry sorting. T_EM_ had reduced precursor frequency but increased total numbers. Our data confirm and expand previous studies that demonstrated that the efficacy of TCR transgenic T cells was not impaired by dexamethasone treatment [43]. Here, endogenously generated anti-tumor immunity could be maintained during dexamethasone treatment if CTLA-4 blockade was provided. Blocking CTLA-4, but not PD-1, partially rescued T cell proliferation in the presence of dexamethasone in vitro. These findings may reflect that CTLA-4 blockade acts upon less differentiated T cells, which are most sensitive to dexamethasone. Systemic immunity elicited by CTLA-4 blockade has been previously shown to promote anti-tumor immunity against melanoma metastases within the central nervous system, indicating that CTLA-4 blockade functions outside of the CNS [15, 44]. The GL261 luciferase-mCherry tumor cells provided an immunogenic intracranial tumor model. By waiting one week before dexamethasone treatment, tumor surveillance and immune activation were permitted, potentially leading to differentiation of tumor-reactive T cells before dexamethasone exposure. In this model, CTLA-4 blockade was sufficient to provide a survival advantage to dexamethasone-treated mice. In contrast, mice exposed to dexamethasone prior to tumor implantation and antigen exposure were unresponsive to CTLA-4 blockade. Collectively, these data suggest that tipping the immune response to more differentiated subsets may lesson the immune suppression imposed by dexamethasone.

These results demonstrate that the timing of dexamethasone treatment relative to the development of anti-tumor immunity significantly impacts the efficacy of immunotherapy. Previous work has shown that corticosteroids provided to alleviate irAEs did not impact the overall response rate of patients with melanoma who received nivolumab [45]. For those patients, corticosteroids were provided after immunotherapy was initiated. In contrast, patients with intracranial tumors are routinely provided high-dose corticosteroids from the period of initial diagnosis until chemoradiation completion, a period that can span 8–12 weeks. Corticosteroids are also provided during surgical resection, as they have been shown to extend survival in this context [46]. Thus, for patients with intracranial tumors, corticosteroids provided before initiation of immunotherapy, a time when the immune system is unlikely to be actively proliferating, may blunt the generation of an anti-tumor response.

Similarly, dexamethasone provided to immunologically “cold” tumors or those with insufficient anti-tumor immunity will likely abrogate new priming and differentiation of anti-tumor T cells. However, the concurrent use of CTLA-4 blockade can encourage T_N_ activation, thereby contributing to antigen spread. Once anti-tumor immunity has been initiated, the negative impact of corticosteroids on immune function is markedly reduced. These results may have important implications in designing future immunotherapy strategies helping to optimize clinical trials for patients with brain cancers as well as other diseases where corticosteroid use is common.

Steroid alternatives may need to be considered for patients with intracranial tumors who wish to enroll on immunotherapy trials. For example, blockade of vascular endothelial growth factor by bevacizumab reduces edema by normalizing tumor vasculature. Further, it has been shown to promote lymphocyte infiltration into the tumor and increase circulating memory T cell numbers [47]. Such alternative approaches may be needed to manage symptoms in patients with intracranial tumors while preserving the potential for anti-tumor immunity.

## Conclusions

Here, we interrogated the impact of dexamethasone on T cell subsets in the setting of immunotherapy. Dexamethasone blocks naïve T cell proliferation and differentiation by attenuating CD28 co-stimulation. Because co-stimulation is essential for successful T cell priming and expansion, these data suggest that corticosteroids impair response in immunotherapy treatment-naïve patients or those with poorly antigenic tumors. However, T cells may be partially protected or rescued from the immunosuppressive effects of dexamethasone with administration of CTLA-4 blockade. Additionally, negative corticosteroid effects are diminished after developing a successful anti-tumor immune response.

## Additional files

Additional file 1: **Figure S1.** T cell stimulated with αCD3/αCD28 microbeads proliferate in the presence of dexamethasone.Healthy donor T cells were cultured for four days with the indicated ratio of αCD3/αCD28 microbeads:total T cells in the presence of vehicle or dexamethasone. **A,** Representative flow cytometry plots of CellTrace violet dilution. Plots were derived from gated CD4 (top row) or CD8 (bottom row) T cells. **B-D,** Proliferation analyses of CD4 T cells (top) and CD8 T cells (bottom) performed on the samples shown in (**A**). Precursor Frequency (**B**), Expansion Index (**C**), and Proliferation Index (**D**) are shown. Samples were plated in duplicate and analyzed with an unpaired students T test. Data are representative of three independent experiments. (PDF 3563 kb)

Additional file 2: **Figure S2. A,** Negatively-selected healthy donor T cells were cultured with the indicated microbeads and vehicle or dexamethasone. The percent of apoptotic CD4 (top) and CD8 (bottom) T cells was assessed by Annexin V/PI. Data are representative of four independent experiments. **B,** Lysates from healthy donor T cells incubated with the indicated microbeads and vehicle or dexamethasone were probed for the indicated proteins. GAPDH was used as a loading control. (PDF 693 kb)

Additional file 3: **Figure S3.** T cell differentiation subsets formed during in vitro stimulation with αCD3/CD80 stimulation. Negatively-selected healthy donor T cells were cultured with 5 μg/mL αCD3 and the indicated concentration of CD80. T cell differentiation subsets were quantified following four days of culture. **A,** Flow plot of gating strategy to identify the indicated T cell differentiation subsets. **B,** Flow plots of CD4 (top) and CD8 (bottom) T cells cultured under the indicated conditions. (PDF 3995 kb)

Additional file 4: **Figure S4.** Increased co-stimulation ameliorates the inhibitory effects of dexamethasone. Negatively-selected healthy donor T cells were cultured with 5 μg/mL αCD3 and increasing concentrations of CD80 in the presence of vehicle or dexamethasone. **A-B.** CD8 T cells cultured with vehicle (**A**) or dexamethasone (**B**). Flow cytometry plots showing proliferation of cells cultured with the indicated concentration of CD80 (left) and total numbers of naïve (T_N_), central memory (T_CM_), effector memory (T_EM_), and terminal effector (T_TE_) T cells following four days of culture (right) are shown. Differentiation subsets were assessed by CD45RO and CCR7 staining. Each condition was plated in duplicate, and data are representative of three independent experiments. Data were analyzed with an unpaired, two-tailed T Test. (PDF 2573 kb)

Additional file 5: **Figure S5** PD-1 blockade does not rescue dexamethasone-mediated proliferation defects. **A,** Flow cytometry analysis of PD-1 surface expression on CD4 (left) or CD8 (right) T cells stimulated with αCD3/αCD28 microbeads. Unstimulated (dashed line), stimulated in presence of vehicle (solid line), and stimulated in presence of dexamethasone (filled red line) are shown. **B,** Geometric median fluorescence intensity (gMFI) of PD-1 staining on CD4 or CD8 T cells. Cells cultured with vehicle (black bars) and dexamethasone (red bars) are shown. Data are an average of duplicate samples. **C,** Expression of PD-1 by qPCR of T cells stimulated in the presence of vehicle or dexamethasone. Data are representative of four independent experiments. **D-E.** Healthy donor T cells were stimulated for four days in the presence of vehicle or dexamethasone and nivolumab or ipilimumab F(ab’)_2_ antibody as indicated. Precursor frequency of CD4 and CD8 T cells was quantified by FlowJo. The ratio of dexamethasone to vehicle for CD4 (C) and CD8 (D) T cells is shown. All samples were plated in duplicate and the ratios were analyzed with a one-way ANOVA. Data are representative of *n* = 4 healthy donors. (PDF 2522 kb)

Additional file 6: **Figure S6** CTLA-4 blockade does not rescue dexamethasone pre-treated mice. **A,** Schema of survival experiment. Albino C57Bl/6 mice received intracranial implantation of GL261 ffluc-mCherry glioma cells. Dexamethasone was initiated one day prior to tumor implantation (dex (D-1)) or one week following tumor implantation (dex (D7)). CLTA-4 blockade or isotype antibody were injected on days 13, 16, and 19 following tumor implantation. Mice were randomized on day 6 following tumor implantation into groups of equivalent tumor luminescence. **B,** Kaplan Meier survival curves of mice receiving the indicated treatments. *n* = 8 to 9 mice per cohort. Data are representative of two independent experiments. (PDF 1525 kb)

Additional file 7: **Figure S7.** Quantification of Treg and checkpoint molecules in tumor-bearing mice. GL261 ffluc-mCherry tumor-bearing mice were randomized into the indicated cohorts based on bioluminescence values from tumor. Vehicle or dexamethasone treatment was initiated on day 7, and isotype or CTLA-4 blocking antibody were administered on days 13, 16, and 19 following tumor implantation. Mice were euthanized on day 23 and tissues were harvested for flow cytometry analysis. **A,** Treg cell number from tumor-bearing brain hemisphere (left; n = 8) or the cervical tumor-draining lymph nodes (right; *n* = 10). **B,** The percentage of CD4 (top two plots) or CD8 (bottom two plots) T cells expressing the indicated checkpoint molecules. Co-expression of molecules was quantified using a Boolean gating strategy. Data were analyzed using a unpaired students T test. (PDF 1891 kb)

## Abbreviations

APC: Antigen-Presenting Cell
EI: Expansion Index
irAE: immune-related Adverse Events
PF: Precursor Frequency
PI: Proliferation Index
Tcm: Central Memory T cells
Tem: Effector Memory T cells
Tn: naïve T cells
