# Supplementary material for: Dexamethasone-induced immunosuppression: mechanisms and implications for immunotherapy
Source: J Immunother Cancer. 2018 Jun 11;6:51. doi: 10.1186/s40425-018-0371-5 (PMC5996496; doi:10.1186/s40425-018-0371-5)

Supplementary Figure S1.
T cell stimulated with αCD3/αCD28 microbeads proliferate in the presence of dexamethasone

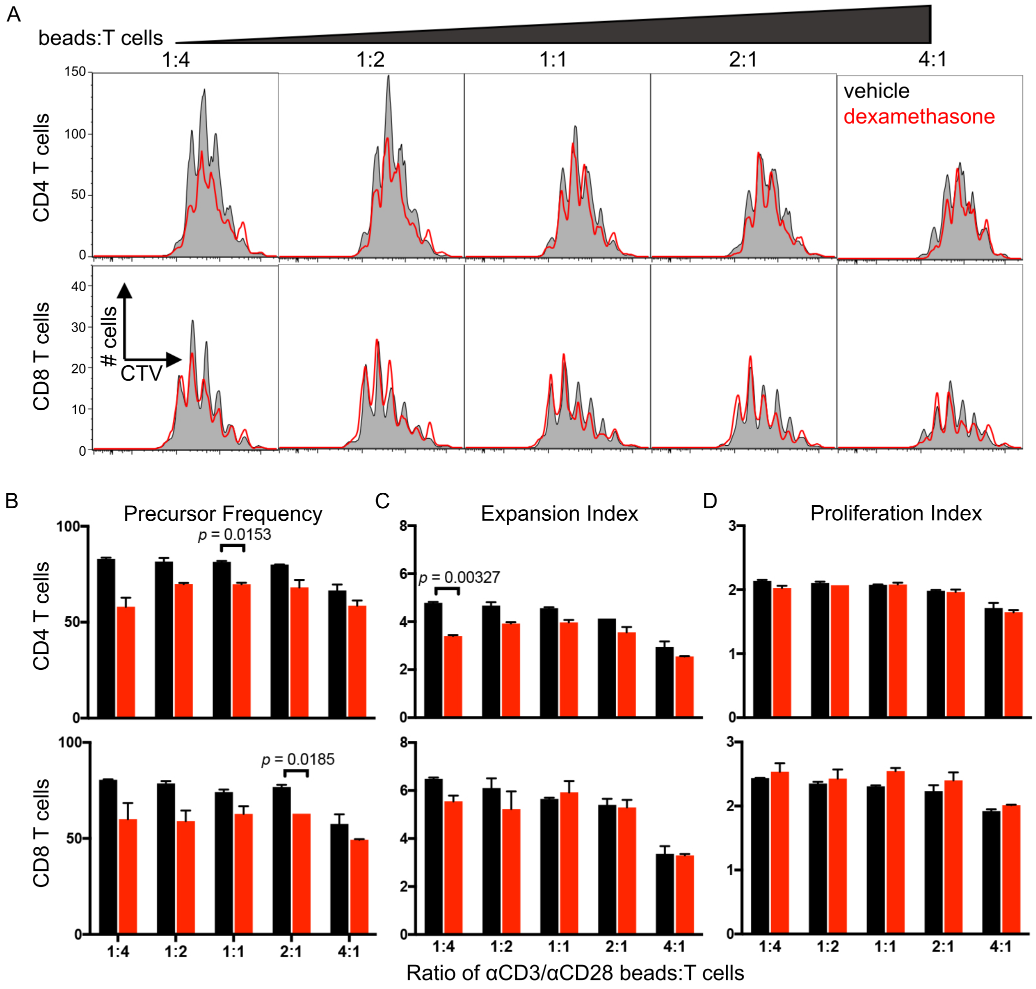

Supplement: Supplementary file 1 — Figure S1. T cell stimulated with αCD3/αCD28 microbeads proliferate in the presence of dexamethasone.Healthy donor T cells were cultured for four days with the indicated ratio of αCD3/αCD28 microbeads:total T cells in the presence of vehicle or dexamethasone. A, Representative flow cytometry plots of CellTrace violet dilution. Plots were derived from gated CD4 (top row) or CD8 (bottom row) T cells. B-D, Proliferation analyses of CD4 T cells (top) and CD8 T cells (bottom) performed on the samples shown in (A). Precursor Frequency (B), Expansion Index (C), and Proliferation Index (D) are shown. Samples were plated in duplicate and analyzed with an unpaired students T test. Data are representative of three independent experiments. (PDF 3563 kb) [file 40425_2018_371_MOESM1_ESM.pdf]
