# Supplementary figures and images for: Dexamethasone-induced immunosuppression: mechanisms and implications for immunotherapy
Source: J Immunother Cancer. 2018 Jun 11;6:51. doi: 10.1186/s40425-018-0371-5 (PMC5996496; doi:10.1186/s40425-018-0371-5)

**Supplementary Figure S2.**  
**Dexamethasone does not induce apoptosis of stimulated T cells**

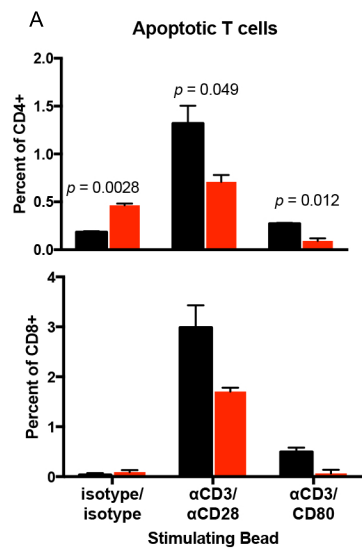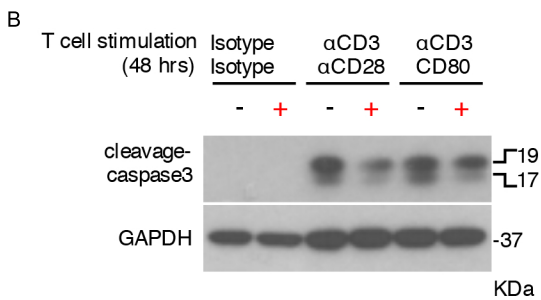

Supplement: Supplementary file 2 — Figure S2. A, Negatively-selected healthy donor T cells were cultured with the indicated microbeads and vehicle or dexamethasone. The percent of apoptotic CD4 (top) and CD8 (bottom) T cells was assessed by Annexin V/PI. Data are representative of four independent experiments. B, Lysates from healthy donor T cells incubated with the indicated microbeads and vehicle or dexamethasone were probed for the indicated proteins. GAPDH was used as a loading control. (PDF 693 kb) [file 40425_2018_371_MOESM2_ESM.pdf]

**Supplementary Figure S3**  
**T cell differentiation subsets formed during *in vitro* stimulation**

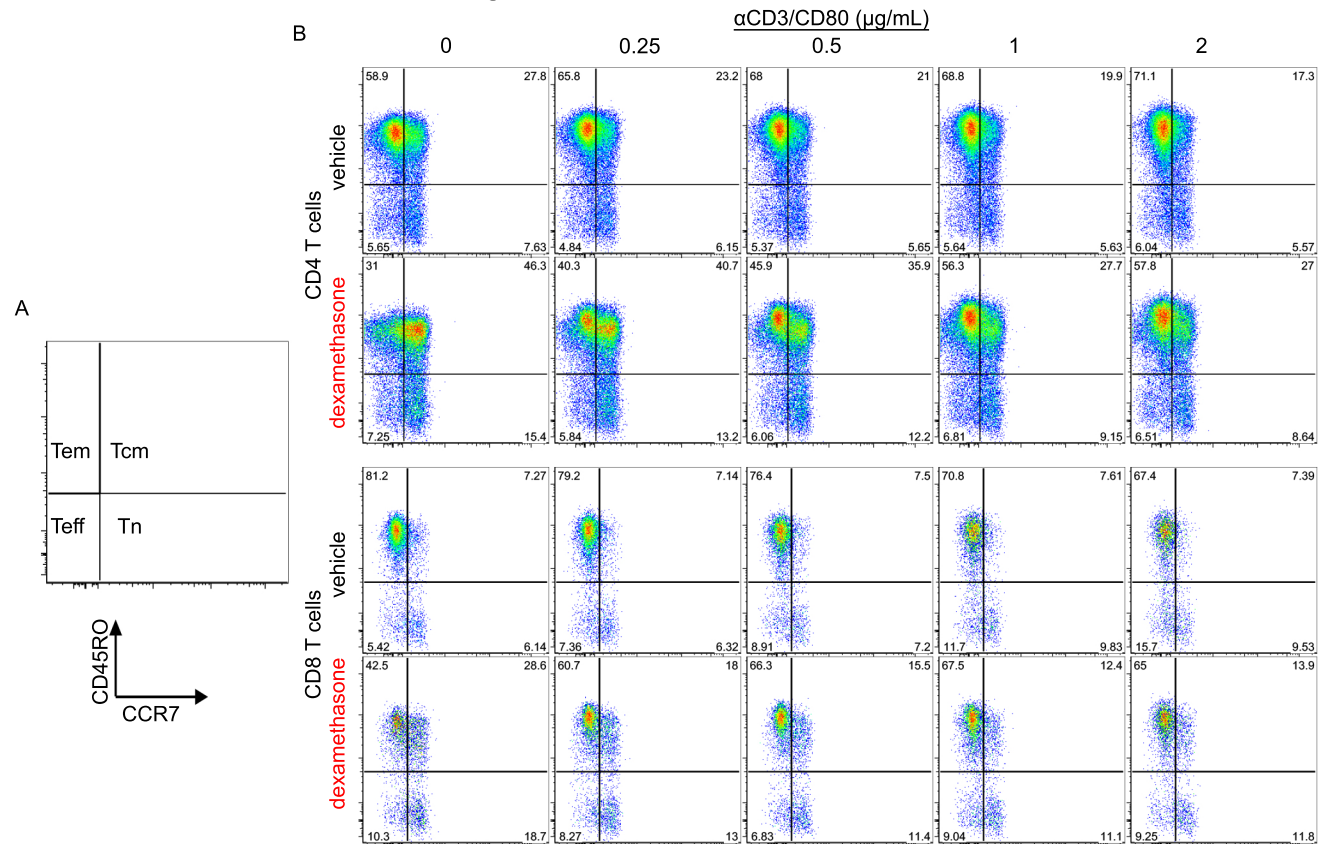

Supplement: Supplementary file 3 — Figure S3. T cell differentiation subsets formed during in vitro stimulation with αCD3/CD80 stimulation. Negatively-selected healthy donor T cells were cultured with 5 μg/mL αCD3 and the indicated concentration of CD80. T cell differentiation subsets were quantified following four days of culture. A, Flow plot of gating strategy to identify the indicated T cell differentiation subsets. B, Flow plots of CD4 (top) and CD8 (bottom) T cells cultured under the indicated conditions. (PDF 3995 kb) [file 40425_2018_371_MOESM3_ESM.pdf]

**Supplementary Figure S6**  
**CTLA-4 blockade does not rescue dexamethasone pre-treated mice**

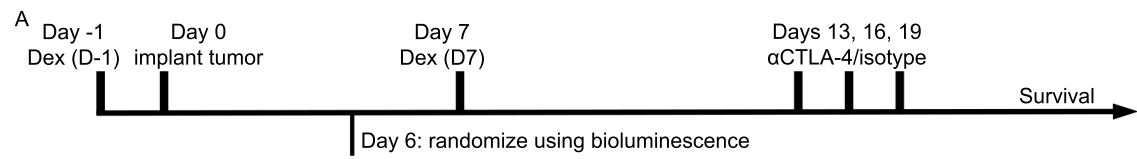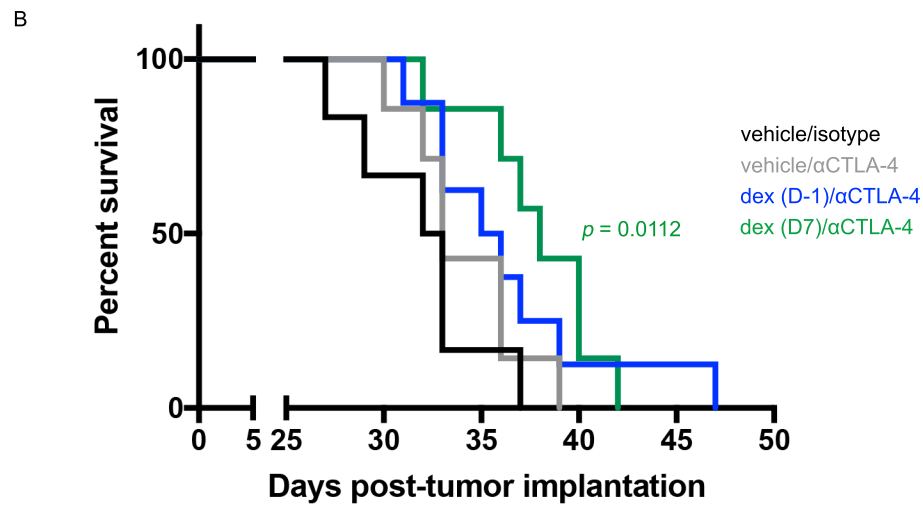

Supplement: Supplementary file 6 — Figure S6 CTLA-4 blockade does not rescue dexamethasone pre-treated mice. A, Schema of survival experiment. Albino C57Bl/6 mice received intracranial implantation of GL261 ffluc-mCherry glioma cells. Dexamethasone was initiated one day prior to tumor implantation (dex (D-1)) or one week following tumor implantation (dex (D7)). CLTA-4 blockade or isotype antibody were injected on days 13, 16, and 19 following tumor implantation. Mice were randomized on day 6 following tumor implantation into groups of equivalent tumor luminescence. B, Kaplan Meier survival curves of mice receiving the indicated treatments. n = 8 to 9 mice per cohort. Data are representative of two independent experiments. (PDF 1525 kb) [file 40425_2018_371_MOESM6_ESM.pdf]
