# Supplementary material for: Dexamethasone-induced immunosuppression: mechanisms and implications for immunotherapy
Source: J Immunother Cancer. 2018 Jun 11;6:51. doi: 10.1186/s40425-018-0371-5 (PMC5996496; doi:10.1186/s40425-018-0371-5)

Supplementary Figure S4
Increased co-stimulation ameliorates inhibitory effects of dexamethasone

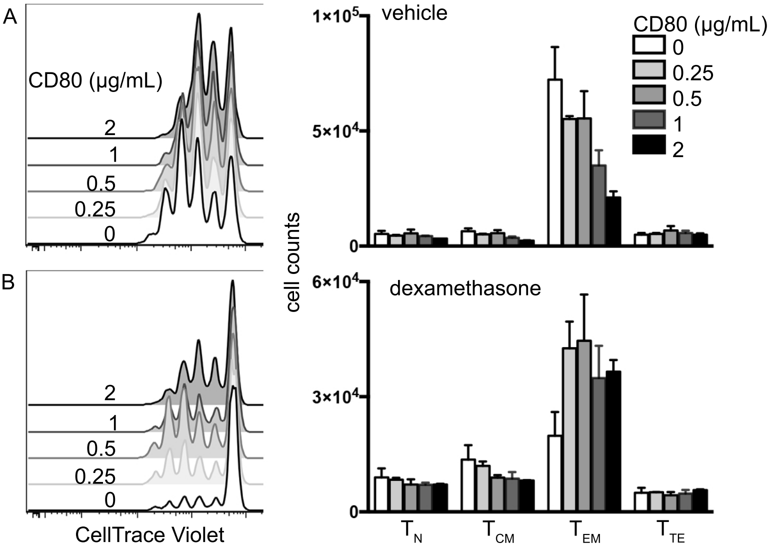

Supplement: Supplementary file 4 — Figure S4. Increased co-stimulation ameliorates the inhibitory effects of dexamethasone. Negatively-selected healthy donor T cells were cultured with 5 μg/mL αCD3 and increasing concentrations of CD80 in the presence of vehicle or dexamethasone. A-B. CD8 T cells cultured with vehicle (A) or dexamethasone (B). Flow cytometry plots showing proliferation of cells cultured with the indicated concentration of CD80 (left) and total numbers of naïve (TN), central memory (TCM), effector memory (TEM), and terminal effector (TTE) T cells following four days of culture (right) are shown. Differentiation subsets were assessed by CD45RO and CCR7 staining. Each condition was plated in duplicate, and data are representative of three independent experiments. Data were analyzed with an unpaired, two-tailed T Test. (PDF 2573 kb) [file 40425_2018_371_MOESM4_ESM.pdf]
