# Supplementary material for: Dexamethasone-induced immunosuppression: mechanisms and implications for immunotherapy
Source: J Immunother Cancer. 2018 Jun 11;6:51. doi: 10.1186/s40425-018-0371-5 (PMC5996496; doi:10.1186/s40425-018-0371-5)

Supplementary Figure S5

PD-1 blockade does not rescue dexamethasone-induced proliferation defects

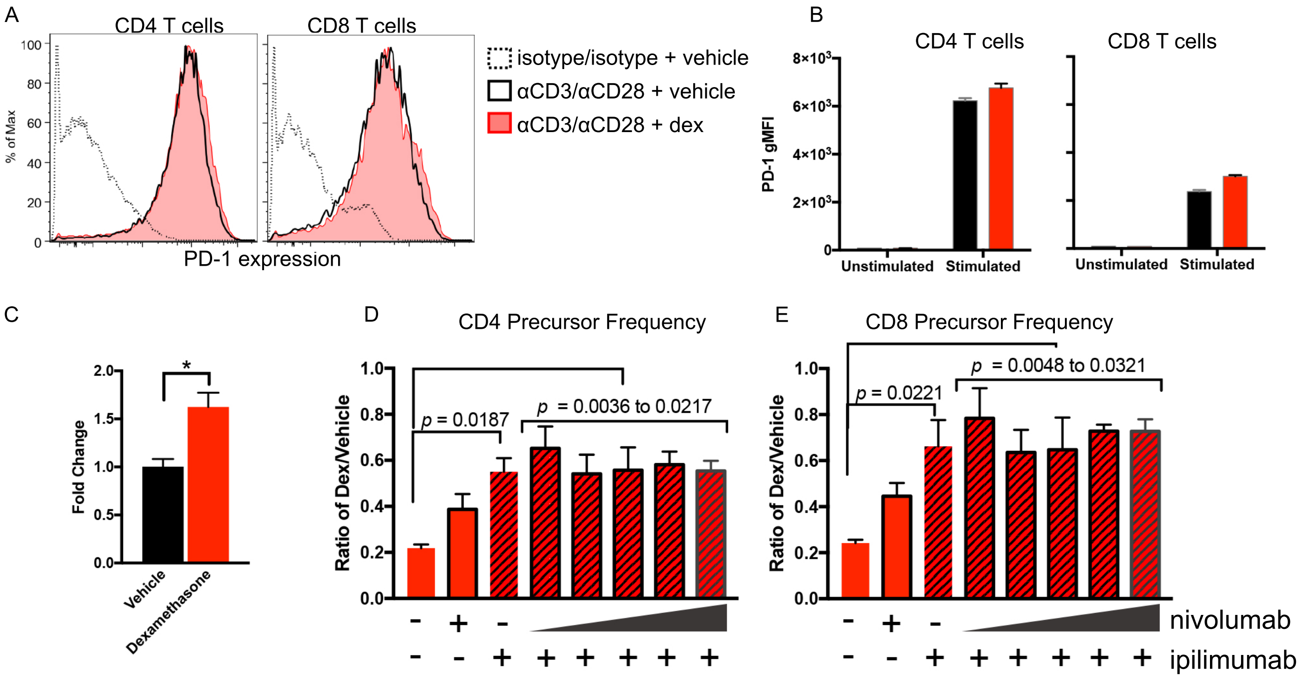

Supplement: Supplementary file 5 — Figure S5 PD-1 blockade does not rescue dexamethasone-mediated proliferation defects. A, Flow cytometry analysis of PD-1 surface expression on CD4 (left) or CD8 (right) T cells stimulated with αCD3/αCD28 microbeads. Unstimulated (dashed line), stimulated in presence of vehicle (solid line), and stimulated in presence of dexamethasone (filled red line) are shown. B, Geometric median fluorescence intensity (gMFI) of PD-1 staining on CD4 or CD8 T cells. Cells cultured with vehicle (black bars) and dexamethasone (red bars) are shown. Data are an average of duplicate samples. C, Expression of PD-1 by qPCR of T cells stimulated in the presence of vehicle or dexamethasone. Data are representative of four independent experiments. D-E. Healthy donor T cells were stimulated for four days in the presence of vehicle or dexamethasone and nivolumab or ipilimumab F(ab’)2 antibody as indicated. Precursor frequency of CD4 and CD8 T cells was quantified by FlowJo. The ratio of dexamethasone to vehicle for CD4 (C) and CD8 (D) T cells is shown. All samples were plated in duplicate and the ratios were analyzed with a one-way ANOVA. Data are representative of n = 4 healthy donors. (PDF 2522 kb) [file 40425_2018_371_MOESM5_ESM.pdf]
