# Supplementary material for: Dexamethasone-induced immunosuppression: mechanisms and implications for immunotherapy
Source: J Immunother Cancer. 2018 Jun 11;6:51. doi: 10.1186/s40425-018-0371-5 (PMC5996496; doi:10.1186/s40425-018-0371-5)

Supplementary Figure S7
Quantification of Treg and checkpoint molecules in tumor-bearing mice

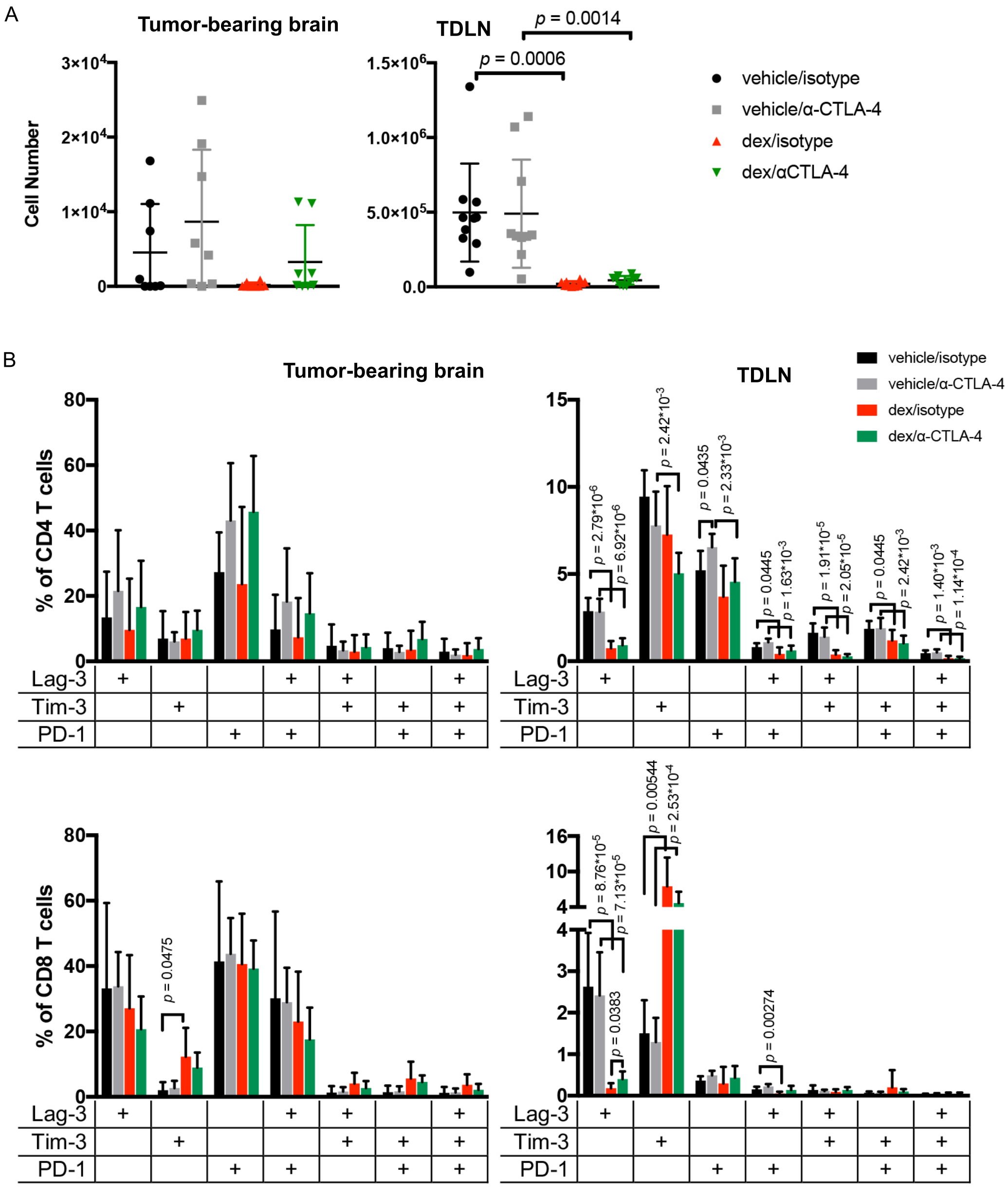

Supplement: Supplementary file 7 — Figure S7. Quantification of Treg and checkpoint molecules in tumor-bearing mice. GL261 ffluc-mCherry tumor-bearing mice were randomized into the indicated cohorts based on bioluminescence values from tumor. Vehicle or dexamethasone treatment was initiated on day 7, and isotype or CTLA-4 blocking antibody were administered on days 13, 16, and 19 following tumor implantation. Mice were euthanized on day 23 and tissues were harvested for flow cytometry analysis. A, Treg cell number from tumor-bearing brain hemisphere (left; n = 8) or the cervical tumor-draining lymph nodes (right; n = 10). B, The percentage of CD4 (top two plots) or CD8 (bottom two plots) T cells expressing the indicated checkpoint molecules. Co-expression of molecules was quantified using a Boolean gating strategy. Data were analyzed using a unpaired students T test. (PDF 1891 kb) [file 40425_2018_371_MOESM7_ESM.pdf]
